# Measuring responsiveness and respectful treatment in maternity care in sub-Saharan Africa: a questionnaire validation and development of a score

**DOI:** 10.1186/s12884-025-07319-3

**Published:** 2025-03-21

**Authors:** Zoë Morris, Soha El Halabi, Claudia Hanson, Bianca Kandeya, Elizabeth Ayebare, Gisele Houngbo, Anastasia Månsson, Fadhlun Alwy Al-Beity, Kristi Sidney Annerstedt

**Affiliations:** 1 Department of Global Public Health, Karolinska Institutet, Solna, Sweden; 2 Department of Disease control, London School of Hygiene & Tropical Medicine, London, UK; 3 Kamuzu University of Health Sciences- Centre for Reproductive Health, Blantyre, Malawi; 4 Centre of Excellence for Maternal Newborn and Child Health, Department of Health Policy Planning and Management, School of Public Health, Makerere University, Kampala, Uganda; 5 Centre de Recherche en Reproduction Humaine et en Démographie (CERRHUD), Benin College of Medicine, Cotonou, Benin; 6 Department of Obstetrics and Gynaecology, Muhimbili University of Health and Allied Sciences, Dar Es Salaam, Tanzania

**Keywords:** Factor analysis, Person-centered care, Psychometric analysis, Respectful maternity care, Sub-Saharan Africa, Validation, Scale score

## Abstract

**Introduction:**

The importance of respectful maternity care on optimal maternal outcomes is increasingly acknowledged globally. However, mistreatment and abuse are still experienced by women during hospital childbirth in many parts of the world, with sub-Saharan Africa being one of the places where it is most common. Interventions aiming to improve respectful maternity care must be able to assess the prevalence of responsiveness and mistreatment women experience. This is usually done with questionnaires, though these are not always validated. Scores to represent the level of responsiveness and mistreatment experienced can be created from questionnaire results and have many uses, though no score is consistently used in this field. A new questionnaire measuring responsiveness and respectful treatment was developed for use in the ALERT project, as a questionnaire covering both of these concepts did not exist. This study aimed to validate this questionnaire and to create a scoring method.

**Methods:**

Psychometric analyses, including exploratory and confirmatory factor analysis, were performed on cross-sectional data collected from the ALERT study to identify and confirm underlying factors. Using these factors, simple summation and factor-weighted methods were used to create scores and their results compared.

**Results:**

Six factors were identified: “Communication & supportive care”, “Hospital environment”, “Maintained respect & dignity”, “Social support”, “Maintained privacy & confidentiality” and “Lack of physical & verbal abuse”. The results of the two scoring methods developed were similar.

**Conclusions:**

The responsiveness and respectful treatment questionnaire has high validity in the ALERT study population for the six factors identified. The two scoring methods developed are useful for different aspects of the ALERT intervention and can be used to facilitate comparisons or measure progress towards improving respectful maternity care in these settings.

**Supplementary Information:**

The online version contains supplementary material available at 10.1186/s12884-025-07319-3.

## Introduction

Women across the world experience mistreatment and abuse during facility-based childbirths [1]. Mistreatment manifests in various forms including disrespect, physical and verbal abuse, poor rapport between women and health providers, stigma, discrimination and failure to meet professional standards of care [1]. Disrespect and abuse are particularly common in maternity services in sub-Saharan Africa, with a systematic review finding that 44% of women reported experiencing these at a healthcare facility during childbirth [2].

Mistreatment is not only a violation of a woman’s right to dignified and respectful healthcare but may also have direct and indirect impacts on the woman and her baby. For instance, fear of abuse may result in women presenting very late to hospital or not seeking antenatal care [3]. Bad experiences may also deter women from giving birth in hospital in the future, which is accompanied by much higher rates of complications for both the woman and the child [3].

Providing high quality maternity care is thus crucial to improving the health of women and their babies. In 2015, the World Health Organization (WHO) proposed a framework for quality of care, underlining the importance of technically competent care as well as women’s experiences of care [4]. Respectful maternity care (RMC), which refers to the “humane and dignified treatment of a childbearing woman throughout her pregnancy, birth, and the period following childbirth”, is a key component of quality of care [5]. RMC also includes the responsiveness of the care received, defined as “a measure of how the health system addresses legitimate expectations of individuals”, including, for instance, their expectations of the care they will receive and the facilities they will use [6]. A growing body of literature has focused on qualitatively understanding RMC and the manifestations of mistreatment, in addition to quantifying the prevalence of mistreatment during facility-based childbirths.

The Bowser and Hill landscape analysis was one of the first attempts to shed light on the issue of mistreatment of women in facilities during childbirth [3]. Bohren et al. developed the categorisation for mistreatment of women during childbirth based on this work [7]. Afulani et al. broadened the perspective to include further dimensions of person-centred care [8], as highlighted in the WHO’s quality of care framework [4], such as communication, respect and dignity, and emotional support. Afulani’s tool was validated in Kenya and India [8, 9]. In 2019, Bohren et al. further highlighted the levels of mistreatment of women during childbirth using two approaches: observations of women throughout the different stages of birth, and community-based surveys administered to women post-partum [10]. Despite this work, there is not an established, standardised methodology for quantitively measuring responsiveness and respectful treatment (or lack of mistreatment), including which type of tool to use and when to administer it, e.g., at discharge or later in the postpartum period.

As part of a maternal health, hospital-based quality improvement intervention project (the ALERT study) being conducted in four sub-Saharan African countries (Benin, Malawi, Tanzania and Uganda), a questionnaire was developed to measure responsiveness and respectful treatment, two secondary outcomes of the study [11]. This new questionnaire was formed from the combination of two well-known questionnaires [8, 12] to create a questionnaire which adequately captured both responsiveness and respectful treatment. Whilst many of the questions were similar across the questionnaires, Afulani’s questionnaire [8] included additional questions about staff demeanour and hospital facilities, and Bohren’s questionnaire [12] asked more detailed questions about abuse received. The questionnaire was administered to women after giving birth, prior to their discharge from the hospital [11].

This newly developed questionnaire needed to be validated in its new form. Few of the existing questionnaires investigating experiences of care during the peripartum period were suitably validated [13]. Questionnaires are often designed to measure concepts, such as responsiveness or respectful treatment, with multiple questions investigating different aspects of these concepts. These concepts, also known as constructs, domains or latent factors, are often difficult to measure directly and therefore it is important to test the validity of questionnaires to ensure that the questions asked are capturing the intended concepts [14]. Assessing validity is particularly important when using a newly developed questionnaire, or when a questionnaire is first administered in a new context, such as in a different country or age group [15]. If a questionnaire does not have high validity, meaningful conclusions about concepts cannot be drawn from the results.

Valid constructs are particularly useful when developing indicator scores. Aggregated scores from questionnaires facilitate easier comparisons, such as between countries, across intervention time points and between studies [10]. Such scoring, especially when based on validated tools, can also be used to increase facilities’ accountability and as a method to track their progress towards goals [10]. Little consensus exists, however, on the best way to create such scores and therefore studies rarely use comparable methodologies [16].

The two main aims of this paper were to (1) evaluate the validity of the responsiveness and respectful treatment questionnaire used in the ALERT study and (2) develop a responsiveness and respectful treatment indicator score. Assessing the validity of the newly developed questionnaire would ensure appropriate use of the ALERT data collected and, if found to have high validity, would contribute a useful assessment tool to the wider RMC research field and future studies. The development of an indicator score would aid in assessing the progress of the ALERT intervention and help harmonise future research and comparisons.

## Methods

### Study design and setting

Cross-sectional data were collected as part of the ALERT (“Action leveraging evidence to reduce perinatal mortality and morbidity in sub-Saharan Africa”) study [11]. The ALERT study includes a four-component intervention: (1) an intervention co-designed with healthcare providers and women, (2) in-service competency-based training for midwives, (3) quality improvement and (4) leadership mentoring for healthcare providers in maternity wards [11]. Improving responsiveness and reducing hospital-based mistreatment are secondary outcomes of the intervention and embedded in the different components [11].

A total of 16 hospitals were included from four countries in sub-Saharan Africa: Benin, Malawi, Tanzania and Uganda. Details regarding the criteria for selecting hospitals are contained in the ALERT study protocol [11]. The hospitals were in peri-urban and rural areas and included a mixture of private-not-for-profit and public hospitals [11]. Data collection took place at each hospital every six months, beginning December 2021, with three rounds of data collection having taken place in each country as of December 2022 and thus included in this analysis. Data were collected by trained data collectors and were entered into the Research Electronic Data Capture (REDCap) system using tablets [17].

### Study participants

Women were recruited after giving birth in one of the study hospitals. The questionnaire was administered to the women by a member of the ALERT research team prior to their discharge. Women who gave birth in the hospital to a baby weighing ≥ 1000 g were eligible to participate. Written, informed consent was obtained from participants before administration of the questionnaire. All consent forms were translated to local languages (French in Benin, Chichewa in Malawi, Swahili in Tanzania, and Luganda in Uganda, with English also being used in Uganda). Trained data collectors (11 women, 3 men) with nursing or social science backgrounds administered the study tool using REDCap tablet-based software. All data collectors received initial training on tool administration and translation validation, followed by refresher sessions before each data collection round.

Fifty women were recruited from each hospital per data collection round. Each data collector obtained a list of eligible women who were to be discharged on the day of data collection. A randomisation factor was applied based on the number of women to be discharged. If 14 or fewer eligible women were to be discharged, they were all interviewed. A randomisation factor of 1:2 (i.e., one of two women) was applied if the number of eligible discharged women was between 15 and 24. A systematic review found that a 20:1 ratio of participant to variable was most accurate for this type of analysis [18]. This suggests that 720 women is an adequate sample size for this analysis.

### Responsiveness and respectful treatment questionnaire

The responsiveness and respectful treatment questionnaire administered was developed by combining two pre-existing questionnaires: The first of these, the Person-Centred Maternity Care (PCMC) scale, was developed and validated by Afulani et al. (published in 2017) [8] and the second, the community-survey tool to assess mistreatment and abuse, was developed by Bohren et al. (published in 2018) [12]. The existing tools were not fully suitable for our study as they lacked validation in our specific context and did not comprehensively address both maternal and newborn health outcomes of interest. To better align with the ALERT trial’s objectives, we adapted and combined elements from both tools, adding clearer accounts of mistreatment and relevant newborn health indicators.

The initial draft of variables included in the questionnaire was created during the first ALERT consortium meeting in February 2020 with a specific working group consisting of social scientists, midwives, obstetricians/gynaecologists and public health specialists. Representatives from each of the four countries were present in the meeting and involved in finalising the questionnaire and response options appropriate in their respective countries. The questions were translated into the aforementioned local languages by a team member and the translations were subsequently reviewed by the local country teams. The translations were tested during data collection training and piloting sessions and small changes were made to the wording of the questions for clarity when appropriate. Thirty-six questions were included in the tool which were answered using three types of responses: Likert scale responses (varying from three-point to seven-point), binary responses (“No”, “Yes”) and categorical responses (“No”, “Yes”, “Don’t know”, “Don’t want to say”). All questions and response options are in Supplementary Material 1.

### Statistical analysis

#### Data management

Exploratory Factor Analysis (EFA) can only be conducted using participants with data present in all variables [19]. Respondents who had more than five questions missing were therefore removed from further testing whilst those with fewer than five missing questions had their values imputed with the median value for the missing question, as recommended for categorical, especially binary, variables [20].

Seventeen questions included responses that could not be placed on a scale from positive to negative response options, which is also necessary for the psychometric analyses. For example, questions that included the response of “Not relevant”, “I did not want to”, “Don’t want to say” or “Don’t know” (e.g., “*Did the provider ask you for permission before carrying out a vaginal examination?”*,* “Were you encouraged to eat and drink during labour?*”, “*Were you held down to the bed forcefully by a provider?*” or “*Did any of the providers or other staff suggest or ask you (or your family) for a bribe*,* informal payment or gift?”*, respectively). All questions with these response options were discussed within the research team and appropriate actions taken. This included replacing some answers with the median and combining some categories (for instance, to the question “*Did you feel providers helped you with your pain?*”, the responses “*I experienced pain*,* but I was not distressed and did not need any treatment*” and “*I did not experience any pain*” were combined into one response of “*I did not need any treatment for pain*” which could then be placed on the continuum for that question). Details of the decisions made can be found in Supplementary Material 2.

Thirteen questions were reverse coded to ensure that all questions were in the same direction with a higher score indicating better treatment [21]. The sample was divided into two subsamples (randomly but equally for each country), one for use in the EFA and the other in the Confirmatory Factor Analysis (CFA).

#### Exploratory factor analysis

Data were considered suitable for EFA if Bartlett’s Test of Sphericity had a p-value ≤ 0.05 and the Kaiser-Meyer-Olkin (KMO) Measure of Sampling Accuracy was ≥ 0.5 [21]. Given that the primary goal of this analysis was exploratory in nature and not data reduction, Common Factor Analysis was used [21, 22]. Principal axis factor (PAF) extraction methods were used to make fewer assumptions regarding data distribution [21] and oblique rotation (Promax) was used to avoid making assumptions regarding the correlation of the questions [23]. Polychoric correlations were used throughout the analyses due to the categorical nature of the responses [24].

Horn’s parallel analysis and a scree plot were used to identify the initial number of factors to test in the EFA [21]. EFA was conducted using this number of factors and the solution assessed using four criteria: (1) no question should load significantly onto more than one factor (cross-loading) (a factor loading, or pattern coefficient, of ≥|0.4| was considered significant [14]), (2) at least three questions should load significantly onto each factor, (3) all factors should have acceptable internal reliability (≥ 0.7) and (4) all factors should make theoretical sense [6]. Leniencies with these criteria however, as suggested by experts, included allowing cross-loading of a few questions (also referred to as items) when there was theoretical justification for why the question was associated with more than one factor [18, 21]. Two-item factors were also not rejected automatically, instead EFA was performed again with fewer factors to see if the two items collapsed onto a factor with other items [14, 21]. If they did not collapse onto another factor then they were considered a valid factor, especially if the items had high factor loadings [18, 23]. McDonald’s omega coefficient [25] was used as an internal reliability estimate [23]. Great weight was put onto the fourth criterion and solutions were not accepted or rejected solely based on the three statistical criteria [14, 23]. Judging the theoretical basis of the solutions was carried out through thorough literature searches and discussions within the research team.

EFA was performed iteratively, with one less factor specified each time, until an acceptable solution was identified [21]. If no acceptable solutions were found, then any potentially problematic questions, such as questions consistently loading onto multiple factors, were excluded and the entire EFA was re-run [21, 24].

Questions which were not part of the final solution, due to not loading sufficiently onto any factors, were reviewed by the research team. If there was strong theoretical reasoning for why the question should be included on a particular factor, it was considered for inclusion in the factor [14]. Questions which justifiably cross-loaded in the final solution were mapped onto their highest loading factor.

#### Confirmatory factor analysis

CFA was performed using the accepted EFA solution on the second subsample of data. Diagonally Weighted Least Squares (DWLS) estimation method was used since the data were ordinal and had few categories [23]. Four measures were used to determine the acceptability of the solution: (1) Comparative Fit Index (CFI) (> 0.9 for acceptable fit, > 0.95 for good fit), (2) Tucker-Lewis Index (TLI) (> 0.9 for acceptable fit, > 0.95 for good fit), (3) Root Mean Square of Approximation (RMSEA) (< 0.08 for acceptable fit, < 0.05 for good fit) and (4) Standardised Root Mean Square Residual (SRMR) (< 0.08 for good fit) [23, 26]. These were not considered strict cut-off values, and a solution need not meet all of the criteria to be accepted, particularly as it has been acknowledged that SRMR performs less well when variables are categorical [14, 23].

These analyses used Stata (version 16.1) [27] and R (version 4.1.2) [28], including the Lavaan [29], psych [30] and EFAtools packages [31].

### Responsiveness and respectful treatment scores

The EFA subscale results were used to create scores of the women’s experiences, with one score per subscale (on a scale of 0–10) [32]. Two methods for creating the score were used and the results compared. Both methods firstly involved normalising, or scaling, the responses to the same scale, with 0 being the worst, or least desirable, response and 1 being the best. This was done by dividing the response value by the number of response options minus one (excluding any “Not relevant”, “Don’t want to say” or “Don’t know” categories) [33]. For instance, if a question had a five-point Likert scale response (0–4), with an additional “Not relevant” category, women who had answered a response on the Likert scale would have their response value divided by four. Women who answered “Not relevant”, “Don’t want to say” or “Don’t know” did not receive values for those questions.

The first method used simple summation [33] and will be referred to as the sum score $\:\left(SS\right)$ here on in [16]. For this method, the normalised responses were summed for each of the questions answered (for which a usable response value was received) in a subscale and divided by the number of questions answered in the subscale (first section of Eq. (1)). Whilst factor loading values were not included in this method, the sign of the question’s pattern factor loading was incorporated, as questions with negative factor loadings needed to be subtracted from the overall score, rather than added [33]. As a result of this, if a negative factor loading was present, then the range of possible sum scores was not 0–10 but began below 0. The second section of Eq. (1) accounted for this and ensured that the range of sum scores was 0–10, whilst maintaining the distribution of the scores. Equation (1) therefore produced a sum score for the latent factor which was comparable across all factors irrespective of the number of questions answered and the factor loadings. In Eq. (1) $\:n$ is the number of questions answered in the subscale, $\:i$ is the index of an answered question in the range of 1 to $\:n$, $\:{w}_{i}$ is the factor loading of question $\:i$ from the EFA solution, $\:{q}_{i}$ is the participant’s response to question $\:i$, and $\:{c}_{i}$ is the number of categories or response options in question $\:i$.

1 $$\:SS=10*\left(\left(\frac{\sum\:_{i=1}^{n}sgn\left({w}_{i}\right)\left(\frac{{q}_{i}}{{c}_{i}-1}\right)}{n}\right)+\left(\frac{{\sum\:}_{i=1}^{n}\varvec{I}\left({w}_{i}<0\right)}{n}\right)\right)$$

In Eq. (1), $\:sgn$ is the sign function such that:

2 $$\:sgn\left( {{w_i}} \right) = \:\left\{ {\begin{array}{*{20}{c}}{ - 1\:\:{\text{if}}\:{w_i} < 0} \\ {\:0\:\:\:\:\:{\text{if}}\:{w_i} = 0} \\ {\:1\:\:\:\:\:{\text{if}}\:{w_i} > 0} \end{array}} \right.$$

and $\:\varvec{I}$ is the indicator function such that:

3 $$I\left( {{w_i} < 0} \right) = \:\left\{ {\begin{array}{*{20}{c}}1&{{\text{if}}}&{{w_i}}&{ < 0} \\ 0&{{\text{if}}}&{{w_i}}&{\:0} \end{array}} \right.$$

The second method used a factor-weighted method to create a factor score $\:\left(FS\right)$ [16, 32]. The normalised question responses were multiplied by their pattern factor loading from the EFA solution [32]. The resulting numbers were then summed for each subscale and that value divided by the sum of the absolute values of the factor loadings of the questions answered in the subscale, as shown in the first section of Eq. (4). The second section of Eq. (4) ensures that the possible range of factor scores is always 0–10 by shifting the score, but keeping the distribution, when a negative factor loading is present in the subscale (see Equation $\:\left(3\right)$ for the definition of $\:\varvec{I}$). In Eq. (4) $\:n$ is the number of questions answered in the subscale, $\:i$ and $\:j$ are the indices of answered questions in the range of 1 to $\:n$, $\:{w}_{i}$ is the factor loading of question $\:i$ from the EFA solution, $\:{q}_{i}$ is the participants response to question $\:i$, $\:{c}_{i}$ is the number of categories or response options in question $\:i$, and $\:\left|{w}_{i}\right|$ and $\:\left|{w}_{j}\right|$ are the absolute factor loadings of questions $\:i$ and $\:j$ from the EFA solution, respectively.

4 $$\:FS=10*\left(\left(\frac{\left.\sum\:_{i=1}^{n}\left({w}_{i}\right)\left(\frac{{q}_{i}}{{c}_{i}-1}\right.\right)}{{\sum\:}_{j=1}^{n}\left|{w}_{j}\right|}\right)+\left(\frac{\sum\:_{i=1}^{n}\varvec{I}\left({w}_{i}<0\right)\left|{w}_{i}\right|}{{\sum\:}_{j=1}^{n}\left|{w}_{j}\right|}\right)\right)$$

Equations (1) and (4) produce scores for individual subscales. It was also thought useful to have a score to represent a woman’s experience of responsiveness and respectful treatment overall. This overall score $\:\left(OS\right)$ was represented as a percentage of the ideal treatment, or best practice (100%), that a woman should receive. To do this the scores ($\:SS$ or $\:FS$) for each of the subscales which received a score were first summed (note that if a subscale had all missing values, a score was not created for that subscale). This summed value was then divided by the number of subscales for which a score was calculated and then multiplied by 10 to produce a percentage. This is shown in Eq. (5) where $\:N$ is the number of subscales which received a score, $\:k$ is the index of a subscale in the range of 1 to $\:N$, and $\:s_k$ is the score that the subscale$\:k$ received. This process was the same for if the subscale score was calculated using the sum or the factor scoring methods.

5 $$\:OS=10*\left(\frac{{\sum\:}_{k=1}^{N}{s}_{k}}{N}\right)$$

In addition, it was thought that having separate indicators for responsiveness and for respectful treatment may be useful. For this reason, if the EFA factors found could clearly and justifiably be separated into factors representing responsiveness and factors representing respectful treatment, two separate scores would be calculated. This would be done using the same method as in Eq. (5) but only including the relevant subscales.

## Results

### Study population

Interviews were collected over three rounds of data collection at all 16 hospitals. The dates of the data collection rounds can be found in Supplementary Material 3. Fifteen people had missing values. One of these women was removed due to having more than 50% missing answers and the remaining 14 women had their missing values imputed using the median of the respective variables.

**Fig. 1:**
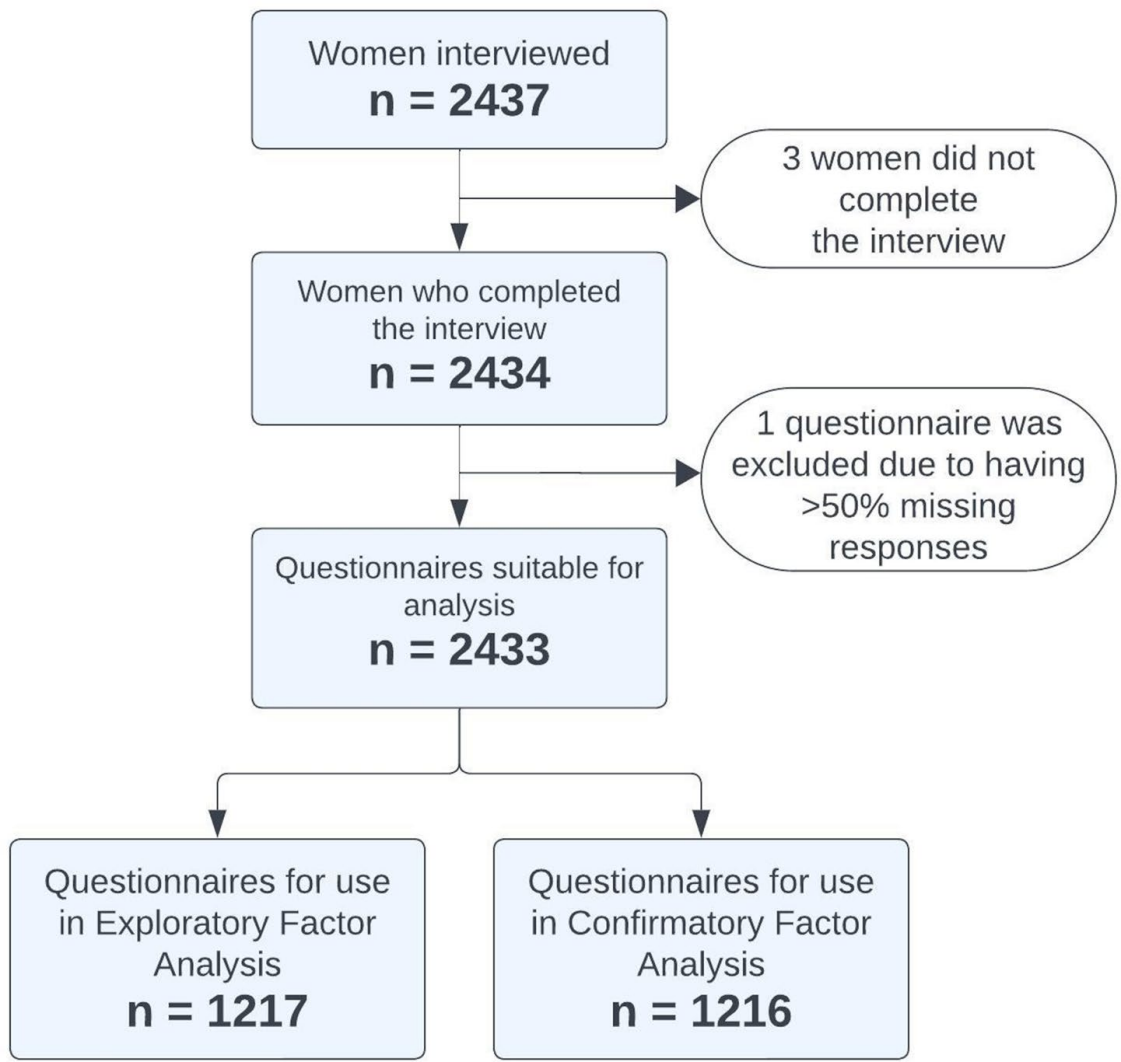
A flow chart showing the number of women involved in each stage of the analysis. Reasons for exclusion are shown in the white boxes. In the final stage the sample was split randomly, though equally for each country, to use half for each type of factor analysis

The final sample size for the analyses was 2433 women (Fig. 1). This sample was divided into two subsamples, comprising of 1217 women for the EFA analysis and 1216 for the CFA analysis. Demographic information and relevant information are detailed in Table 1.

**Table 1:** Characteristics of the final study population

Characteristic	Benin (*n* = 606)		Malawi (*n* = 604)		Tanzania (*n* = 618)		Uganda (*n* = 605)	
**Mean age** (years)	27.1 (SD = 5.9)		24.2 (SD = 6.2)		26.4 (SD = 7.0)		25.2 (SD = 6.1)	
	**No.**	**%**	**No.**	**%**	**No.**	**%**	**No.**	**%**
**Data collection round**								
1	201	33.2%	204	33.8%	210	34.0%	200	33.1%
2	203	33.5%	200	33.1%	204	33.0%	203	33.6%
3	202	33.3%	200	33.1%	204	33.0%	202	33.4%
**Birth outcome**								
Alive	576	95.1%	590	97.7%	604	97.7%	575	95.0%
Stillbirth	30	5.0%	14	2.3%	14	2.3%	30	5.0%
**Mode of birth**								
Vaginal^1^	329	54.3%	515	85.3%	407	65.9%	431	71.1%
Caesarean section	277	45.7%	89	14.7%	211	34.1%	174	28.7%

^1^ “Vaginal” includes: Spontaneous vaginal, Vacuum extraction, Assisted breech and Forceps

SD: Standard deviation

Nb. Percentages may not sum to exactly 100% due to rounding

### Exploratory factor analysis

The data were appropriate for factor analysis based on Bartlett’s Test of Sphericity (*p* < 0.05) and the KMO Measure of Sampling Adequacy (KMO = 0.825).

An 11-factor solution was suggested by Horn’s parallel-analysis factor extraction method and a scree plot. This solution was, however, not accepted as it violated many of the criteria for acceptance; six factors had less than three questions loaded onto them and five questions loaded onto more than one factor. A 10-factor solution was therefore tried which also violated the criteria. Factors were continuously removed until a four-factor solution was tried, which was also not acceptable.

The question which was initially eliminated was regarding forceful pressure (“*Were you held down to the bed forcefully by a provider?*”) as this question consistently cross-loaded, regardless of how few factors were specified, and no theoretical justification for this could be suggested. EFA was therefore re-run with this question removed. The data were still appropriate for factor analysis (Bartlett test < 0.05, KMO = 0.826) however, despite this question being removed, no acceptable EFA solution was found. Another question was then eliminated for the same reasons mentioned previously (“*Did the provider speak to you in a language you could understand?*”). This language question was removed from the original EFA (reintroducing the forceful pressure question) (Bartlett test < 0.05, KMO = 0.830) but an acceptable solution was still not found. However, when these two questions were removed in combination (Bartlett test < 0.05, KMO = 0.831), an acceptable solution was found with six factors. These factors were “Communication & supportive care”, “Hospital environment”, “Maintained respect & dignity”, “Social support”, “Maintained privacy & confidentiality” and “Lack of physical & verbal abuse”. This solution, along with the included questions, their pattern factor loadings and omega internal reliability values are shown in Table 2.

Two factors (“Social support” and “Maintained privacy & confidentiality”) consistently only had two questions loading onto them, but with very high loadings. These questions were not reasonably forced onto other factors when fewer factors were tried. The two-item groupings of these questions made theoretical sense and there were no additional questions that were thought to be related to these factors. It was therefore considered logically sound for these factors to map onto only two questions.

Internal reliability (omega) was ≥ 0.7 for all factors in the final solution apart from the “Lack of physical & verbal abuse” factor. As this omega value was close to 0.7 (0.678), it was accepted as a solution.

**Table 2:** Final exploratory factor analysis solution for the responsiveness and respectful treatment questionnaire

Factor name (ω internal reliability value) Question	Pattern factor loading	
**Communication & supportive care (ω = 0.916)**		
Did the providers introduce themselves to you with their names when they first came to see you?	0.55	
Did you feel the providers explained to you what had been done to you?	0.82	
Did the provider ask you for permission before carrying out a vaginal examination?	0.82	
Did the provider explain to you why they were carrying out examinations or procedures?	0.86	
Did the provider explain to you why they were giving you any medicine?	0.84	
Did you feel you could ask the provider at the hospital any questions you had?	0.66	
Did the provider at the hospital talk to you about how you were feeling?	0.78	
Did the provider at the hospital address your anxieties and fears?	0.51	
Were you encouraged to walk around during labour?	0.53	
Were you encouraged to eat and drink during labour?	0.50	
When you needed help, did you feel the providers at the hospital paid attention?	0.55	
**Hospital environment (ω = 0.735)**		
Would you say the hospital was clean?	0.54	
Was there clean water in the hospital, e.g., for taking a shower?	0.88	
Were you able to access clean drinking water whenever you needed it?	0.91	
**Maintained respect & dignity (ω = 0.814)**		
Did the providers at the hospital treat you with respect?	0.45	
Did the providers at the hospital treat you in a friendly manner?	0.45	
How would you describe the waiting time before you were admitted to the labour ward?	0.53	
Were you shouted or screamed at by a provider or other member of staff?	0.83	
Were you mocked at by a provider or other member of staff?	1.09	
Did you feel the providers at the hospital took the best care of you that they could?	0.46	
Would you recommend a family member to give birth in the same hospital?	0.45	
**Social support (ω = 0.702)**		
Were you allowed to have someone you wanted (family/friend) to stay with you during labour and contractions (1st stage of labour, the time before pushing)?	0.85	
Were you allowed to have someone you wanted (family/friend) to stay with you during birth (2nd stage of labour, pushing)?	0.94	
**Maintained privacy & confidentiality (ω = 0.710)**		
My private or personal information was shared without my consent	1.02	
My physical privacy was violated e.g., being uncovered or having people in the delivery room without my consent	0.67	
**Lack of physical & verbal abuse (ω = 0.678)**		
Were you slapped or pinched by a provider?	0.40	
Did a provider make any negative comments e.g., about age/marital status/ethnicity/religion/HIV status?	0.66	
Were you shouted at or told off because you did not bring items with you?	0.78	
Did any of the providers or other staff suggest or ask you (or your family) for a bribe, informal payment or gift?		-0.73

ω: McDonald’s Omega coefficient

Seven questions did not load onto the final accepted solution, with five not having high enough loadings and two being the questions intentionally removed to achieve the accepted solution. These questions are listed in Table 3.

**Table 3:** Questions which were not included in the final Exploratory Factor Analysis solution

Question	Factor loading on the highest factor
Did the providers call you by your name?	0.35: Hospital environment
Did you have forceful downwards pressure placed on your abdomen before the baby came out?	0.32: Hospital environment
During labour and childbirth, did you feel like you were able to be in the position of your choice?	0.35: Social support
Did you feel providers helped you with your pain?	0.37: Maintained respect & dignity
In general, did you feel safe in the hospital?	0.31: Communication & supportive care
**Intentionally removed**	
Were you held down to the bed forcefully by a provider?	
Did the provider speak to you in a language you could understand?	

### Confirmatory factor analysis

CFA was performed with the EFA solution shown in Table 2. The model fit results were: CFI = 0.949, TLI = 0.943, RMSEA = 0.072, SRMR = 0.123. The CFI, TLI and RMSEA values all indicated an acceptable fit of the model. The SRMR did not meet the threshold for acceptable fit but, for reasons mentioned in the methods section, it was decided that this CFA supports and confirms the EFA solution in this population.

### Responsiveness and respectful treatment score

The means of the two methods were similar for all of the subscales (Table 4). The subscale of “Lack of physical & verbal abuse” had the greatest difference (0.32) between the two methods.

**Table 4:** Comparison of subscales using two scoring methods: sum score and factor weighted score (range 0–10)

Subscale	Method			
	Sum score (SS)		Factor score (FS)	
	Mean	SD	Mean	SD
Communication & supportive care	5.47	2.42	5.58	2.51
Hospital environment	7.63	2.39	7.69	2.57
Maintained respect & dignity	8.67	1.44	8.90	1.34
Social support	2.84	3.76	2.81	3.75
Maintained privacy & confidentiality	9.74	1.15	9.74	1.16
Lack of physical & verbal abuse	7.55	0.85	7.23	0.91

SD: Standard Deviation

It was decided by the research team that the subscales could clearly be separated into responsiveness and respectful treatment, with “Communication & supportive care”, “Hospital environment” and “Social support” representing responsiveness, and “Maintained respect & dignity”, “Maintained privacy & confidentiality” and “Lack of physical & verbal abuse” representing respectful treatment. The means and standard deviations were very similar between the two scoring methods for these two indicators and for the overall score (Table 5).

**Table 5:** Comparison of responsiveness, respectful treatment and overall scores created using two scoring methods: sum score and factor weighted score. Women’s experiences were calculated as a percentage of the best practice, with 100% being the optimal experience and treatment that someone would have at the maternity facility and 0% being the worst possible experience

	Method			
	Sum score		Factor score	
	Mean %	SD	Mean %	SD
Responsiveness (% of best practice)	54.38	18.40	54.86	18.74
Respectful treatment (% of best practice)	86.52	7.13	86.52	7.13
Overall score (% of best practice)	70.62	10.58	70.72	10.59

SD: Standard Deviation

## Discussion

We assessed the validity of the responsiveness and respectful treatment questionnaire used in the ALERT study. The results from this psychometric analysis indicated that the responsiveness and respectful treatment questionnaire had high validity in measuring six constructs: “Communication & supportive care”, “Hospital environment”, “Maintained respect & dignity”, “Social support”, “Maintained privacy & confidentiality” and “Lack of physical & verbal abuse”. Seven of the 36 questions in the questionnaire were extraneous to this solution. The two different methods to generate the score yielded similar results.

Due to the novel nature of this questionnaire, our factors cannot be directly compared with previous validation studies using the PCMC or other questionnaires, however there are some overlaps. Initial validations of the PCMC found three domains: Communication, Autonomy and Supportive care [8]. The majority of questions from the PCMC that were used in the ALERT study questionnaire map onto the “Communication & supportive care” or “Maintained respect & dignity” factors. The questions on “Hospital environment”, “Maintained privacy & confidentiality” and “Social support” previously mapped onto Afulani’s three PCMC domains, however in our analysis they mapped exclusively onto each other and so were made into three separate domains [8]. We feel our results are also supported by a thorough literature review commissioned by the WHO to inform the development of their intrapartum guidelines [34, 35]. This review found that what matters most to women during childbirth was: (1) “a clinically and psychologically safe environment*”*, which we believe corresponds to our two domains “Hospital environment” and “Maintained respect & dignity”, (2) *“*practical and emotional support from birth companions*”*, corresponding to our “Social support” factor, and (3) *“*competent, reassuring, kind clinical staff”, corresponding to our “Communicative & supportive care” factor [34]. It also found that women’s expectations of their childbirth were greatly influenced by societal norms [34].

The category of “Lack of physical & verbal abuse” has not been identified in previous PCMC or similar quantitative validation papers, however these questions consistently loaded together and so we were confident in making it a category. This category included items which we interpreted as referring to ‘normalised abuse’ as defined by Freedman (2014): “behaviour that women consider normal or acceptable but others consider disrespect and abuse, or behaviour that women consider disrespect and abuse but providers do not” [36]. Many qualitative studies report on this topic, for instance showing that some women and midwives consider certain forms of abuse, including slapping and shouting, acceptable when they are done to ensure a safe delivery and good outcome for the baby and mother (for instance slapping the thighs to keep the mother’s legs open to ensure the baby does not become asphyxiated [37]) but unacceptable when they are done “out of malice” [3, 38–41].

Whilst it may appear surprising at first glance that the question “*Were you shouted or screamed at by a provider or other member of staff?*” loaded onto a different factor to the question “*Were you shouted at or told off because you did not bring items with you?*” (“Maintained respect & dignity” and “Lack of physical & verbal abuse” factors, respectively), we consider this an example of the concept of normalised abuse. Previous studies using questionnaires have found that some women reported being shouted at and considered it unacceptable and disrespectful [3, 40] however some women thought it was acceptable for providers to shout if they forget to bring certain items to the hospital [38]. This may also be an example of when women have reported positive birth experiences (and therefore may not answer yes to a broader question they feel suggests a negative experience, such as the first question) and only when asked about specific events did they report having experienced abusive behaviour, as they did not consider it abuse [42].

Another question in the “Lack of physical & verbal abuse” factor was regarding informal payments: “*Did any of the providers or other staff suggest or ask you (or your family) for a bribe*,* informal payment or gift?*”. Informal payments are reported to be commonplace in low- and middle-income countries [43] and encompass a variety of situations [44]. Informal payments can be large sums that are asked for with no explanation, can be added onto the cost of the healthcare visit without informing the payee, or can be requested to help pay for their medicines, equipment or staff wages [44]. Similarly, gifts can range from a substantial amount of money requested by the provider after the birth to a small amount of money given as a “token of appreciation” from the mother or family [46]. Some of these occurrences, in particular paying for equipment or medicines and paying a small amount to show appreciation, are considered more justifiable practices that the mothers may not think of as abuse, or practices that are so common or expected that they are normalised [44].

All four questions which loaded onto the “Lack of physical & verbal abuse” factor were reversed prior to analysis, with a larger value therefore being indicative of “better” treatment, such as no slapping or pinching, no negative comments made, not being shouted or screamed at for forgetting items and not being asked for a bribe, informal payment or gift. The three questions which loaded positively onto the factor (“*Were you slapped or pinched by a provider?*”, “*Did a provider make any negative comments e.g.*,* about age/marital status/ethnicity/religion/HIV status?*” and “*Were you shouted at or told off because you did not bring items with you?*”) indicated that, understandably, better treatment in these three aspects was associated with experiencing no physical and verbal abuse. The question “*Did any of the providers or other staff suggest or ask you (or your family) for a bribe*,* informal payment or gift?*”, however, negatively loaded onto the factor, suggesting that people who reported being asked for a bribe, informal payment or gift (considered as “worse” treatment) experienced less physical and verbal abuse. Various reasons could explain this, for instance, those who are asked for an informal payment or bribe may be the women whom the providers think have means to pay it, thereby being the wealthier people who already receive better treatment [45]. Providers may be less likely to ask poorer women, assuming they could not afford to pay extra, and who they treat less well [45].

### Indicator score

This study aimed to develop a responsiveness and respectful treatment indicator score. Whilst far more complex equations for factor scores have been created [16], for the ALERT project it was important to have a relatively simple but accurate score. The equations presented in this paper are practical for use in many different settings and can be used without the need for advanced software packages. Both methods, creating a sum score and creating a factor score, have advantages and disadvantages [46]. The first method is cruder but simpler and easier to make comparisons with other studies and samples [46]. The second method creates a more accurate score for use within the ALERT sample, by giving more weight to the questions which contributed more to the factor, but is less generalisable, due to potential differences in factor loadings between samples [46]. There was little difference between the results from the two methods. After discussions amongst the research team, it was therefore decided that, for the reasons highlighted above, the ALERT project would use the factor score method for within-study calculations and the sum score method for any comparisons with other study populations.

Two methods of utilising the scores are: (1) the subscale scores can be kept as separate subscales so as to measure specific aspects of care or (2) the subscale scores can be combined to form overall scores which summarise the experiences. These have advantages and disadvantages. Combining the subscales is accompanied by the risk that important differences between the subscales are masked. For instance, “Hospital environment” having a top score (having available and clean water), may mask having a poor “Maintained respect & dignity” score, as these would then cancel out to be an overall average score [32]. One solution to this is to combine similar subscales, as done in this paper by summing subscales representing responsiveness separately to those representing respectful treatment. This reduces, though does not eliminate, the risk of masking differences. Subtle masking is demonstrated by the difference in this paper’s responsiveness and respectful treatment scores (Table 5) where the responsiveness score (54–55%) and the respectful treatment score (87%) averaged to an overall score of 71%. This supports the need to look at experiences on a more detailed level, but does not invalidate or diminish the usefulness of the overall score. Combined results can be particularly valuable for analyses which aim to investigate women’s experiences as a whole.

### Strengths and limitations

Many of the strengths of this validation study lie in the data collection from the ALERT project. For one, the dataset was very large. Both the EFA and CFA had more than adequate sample size, increasing the reliability and precision of the results [26]. Another strength is that the questionnaire was administered on discharge from the hospital, which decreased the potential for recall bias and improved the accuracy of answers. This contrasts with some studies which asked about birth experiences many months later and which acknowledge that this may lead to recall bias [40, 47].

We also made no assumptions about the distribution of the data. It is commonplace for papers to use techniques that are only suitable for data with normal distributions, despite being based on binomial or ordinal data, such as short Likert scales. This can create errors in any further calculations. An example of this is that we normalised the question responses to a scale of 0–1 rather than standardising, which would only be appropriate for normally distributed data. We also used statistical methods for the EFA and CFA which are appropriate for use with non-normal data [23].

A strength of our scoring method was that we divided the subscale sums by the number of questions (in the first method) or the sum of the factor loadings (in the second method) to make sure that all scores were on the same scale (0–10 for individual subscales). Similarly, for the overall scores we divided the sum of the subscales by the number of subscales included to get comparable percentages. Many authors do not do this, instead leaving different maximum scores and ranges for each subscale [15]. Whilst both are valid methods which ultimately include the same information, we feel our method makes the scores easier to interpret and compare.

One limitation with this study was that the facilities where the data were collected may not be generalisable to the whole country. Women were recruited from public or faith-based facilities in Benin, Malawi, Uganda and Tanzania. We did not include women who attended fully private facilities or lower-level facilities e.g., primary or community healthcare centres.

Another limitation of our analysis was in the need to impute values which were missing or not applicable. We feel that our choice of imputation method, imputing onto the median value, involved less risk of significantly changing the distribution of data than methods from previous PCMC papers which involved imputing onto the highest value, presuming the best-case scenario [8]. In addition, the number of missing values was relatively small, and so we do not feel that it will have had a significant impact on our results.

Finally, one limitation of the score was that not all questions contributed equally to the overall score for all people. This is due to women who had missing or not applicable values. Whilst missing values were imputed in the factor analysis, this cannot be done when making new scores as they must be able to be calculated without knowing the median values of the population. Women could not be excluded on the basis of missing values as any women who had not applicable answers, such as those who did not have a vaginal examination or those who did not want to have someone accompany them during labour, would then not be able to have a score. Whilst this means that, for the relatively small percentage of women who had missing values, some subscales contributed more to the overall score than for the people who had no missing values, the overall scores did still represent the experience that the women had at the maternity facility, given all the information they provided.

### Future directions

The methods developed and results found in this paper facilitate further valid analyses within and outside this population. The indicator score created enables easier comparisons to be made. The tool could, for instance, be used to assess the prevalence of responsiveness and respectful treatment in the hospitals. It could also be used not only to assess baseline prevalence but also to monitor and evaluate outcomes of interventions, if administered pre- and post-intervention. Possible confounding factors could also be tested, for example previous studies have suggested that women may be more tolerant of some types of abuse if the baby has a positive outcome [43], which could make outcome a confounding factor.

Additional correlations which could be investigated include the busyness of the hospital, whether it was a public or private hospital and the presence of birthing companions. Midwives reported having less time to spend with women, therefore less time to address anxieties [41], and being less tolerant of behaviours, therefore shouting more often, when the hospital was very busy [43]. Differences in the informal payment culture in public and private hospitals have been reported [47] and some studies have found higher PCMC when male companions are present [48].

## Conclusion

This responsiveness and respectful treatment questionnaire has high validity in the ALERT multi-country sample which enables meaningful comparisons and conclusions to be made using the data. The responsiveness and respectful treatment score will enable valid and reliable comparisons within the ALERT project and facilitate comparisons with other studies.

## Electronic supplementary material

Below is the link to the electronic supplementary material.

Supplementary Material 1

Supplementary Material 2

Supplementary Material 3

## Data Availability

No datasets were generated or analysed during the current study.

## Abbreviations

ALERT: Action leveraging evidence to reduce perinatal mortality and morbidity in sub-Saharan Africa
CFA: Confirmatory Factor Analysis
CFI: Comparative Fit Index
DWLS: Diagonally Weighted Least Squares
FS: Factor Score
KMO: Kaiser-Meyer-Olkin
PAF: Principal Axis Factor
PCMC: Person-Centred Maternity Care
OS: Overall Score
RMC: Respectful Maternity Care
RMSEA: Root Mean Square of Approximation
SRMR: Standardised Root Mean Square Residual
SS: Sum Score
TLI: Tucker-Lewis Index
WHO: World Health Organization

## References

[1] Bohren MA, Vogel JP, Hunter EC, Lutsiv O, Makh SK, Souza JP et al. The mistreatment of women during Childbirth in Health facilities globally: a mixed-methods systematic review. PLoS Med. 2015;12(6). 10.1371/journal.pmed.1001847 PMC4488322 26126110

[2] Kassa ZY, Tsegaye B, Abeje A. Disrespect and abuse of women during the process of childbirth at health facilities in sub-saharan Africa: a systematic review and meta-analysis. BMC Int Health Hum Rights. 2020;20(1). 10.1186/s12914-020-00242-y PMC7487593 32894127

[3] Bowser D, Hill MPHK. Exploring evidence for disrespect and abuse in facility-based Childbirth Report of a Landscape Analysis. USAID-TRAction Project; 2010.

[4] Tunçalp Ö, Were WM, Maclennan C, Oladapo OT, Gülmezoglu AM, Bahl R, et al. Quality of care for pregnant women and newborns - the WHO vision. BJOG. 2015;122(8):1045–9. 25929823 10.1111/1471-0528.13451 PMC5029576

[5] Ministry of Public Health. Respectful Maternity Care Orientation Package for Health Care Providers: Participants Guide. 2017.

[6] Mirzoev T, Kane S. What is health systems responsiveness? Review of existing knowledge and proposed conceptual framework. BMJ Global Health. Volume 2. BMJ Publishing Group; 2017. 10.1136/bmjgh-2017-000486 PMC5717934 29225953

[7] Bohren MA, Oladapo OT, Tunçalp Ö, Wendland M, Vogel JP, Tikkanen M et al. Formative research and development of innovative tools for Better Outcomes in Labour Difficulty (BOLD): study protocol. Reprod Health. 2015;12(1). 10.1186/s12978-015-0028-5 PMC4464989 26006320

[8] Afulani PA, Diamond-Smith N, Golub G, Sudhinaraset M. Development of a tool to measure person-centered maternity care in developing settings: validation in a rural and urban Kenyan population. Reprod Health. 2017;14(1):1–18. 28938885 10.1186/s12978-017-0381-7 PMC5610540

[9] Afulani PA, Diamond-Smith N, Phillips B, Singhal S, Sudhinaraset M. Validation of the person-centered maternity care scale in India. Reprod Health. 2018;15(1). 10.1186/s12978-018-0591-7 PMC6114501 30157877

[10] Bohren MA, Mehrtash H, Fawole B, Maung TM, Balde MD, Maya E, et al. How women are treated during facility-based childbirth in four countries: a cross-sectional study with labour observations and community-based surveys. Lancet. 2019;394(10210):1750–63. 31604660 10.1016/S0140-6736(19)31992-0 PMC6853169

[11] Akuze J, Annerstedt KS, Benova L, Chipeta E, Dossou JP, Gross MM et al. Action leveraging evidence to reduce perinatal mortality and morbidity (ALERT): study protocol for a stepped-wedge cluster-randomised trial in Benin. Malawi Tanzan Uganda. 2021;1–14. 10.1186/s12913-021-07155-z PMC8665312 34895216

[12] Bohren MA, Vogel JP, Fawole B, Maya ET, Maung TM, Baldé MD, et al. Methodological development of tools to measure how women are treated during facility-based childbirth in four countries: Labor observation and community survey. BMC Med Res Methodol. 2018;18(1):1–15. 30442102 10.1186/s12874-018-0603-x PMC6238369

[13] Dencker A, Bergqvist L, Berg M, Greenbrook JTV, Nilsson C, Lundgren I. Measuring women’s experiences of decision-making and aspects of midwifery support: a confirmatory factor analysis of the revised Childbirth Experience Questionnaire. BMC Pregnancy Childbirth. 2020;20(1). 10.1186/s12884-020-02869-0 PMC7137445 32252679

[14] Knekta E, Runyon C, Eddy S. One size doesn’t fit all: using factor analysis to gather validity evidence when using surveys in your research. CBE Life Sci Educ. 2019;18(1):1–17. 10.1187/cbe.18-04-0064 PMC6757227 30821600

[15] Putnick DL, Bornstein MH. Measurement invariance conventions and reporting: the state of the art and future directions for psychological research. Dev Rev. 2016;41:71–90. 27942093 10.1016/j.dr.2016.06.004 PMC5145197

[16] McNeish D, Wolf MG. Thinking twice about sum scores. Behav Res Methods. 2020;52(6):2287–305. 32323277 10.3758/s13428-020-01398-0

[17] Harris PA, Taylor R, Minor BL, Elliott V, Fernandez M, O’Neal L, et al. The REDCap consortium: building an international community of software platform partners. Journal of Biomedical Informatics. Volume 95. Academic Press Inc.; 2019. 10.1016/j.jbi.2019.103208 PMC7254481 31078660

[18] Costello AB, Osborne J. Best practices in exploratory factor analysis: four recommendations for getting the most from your analysis. Res Evaluation Practical Assess Res Evaluation. 2005;10:7.

[19] Osborne JW. Best Practices in Exploratory Factor Analysis. 2014.

[20] Osborne J. Best practices in Data Cleaning: a complete guide to Everything You need to do before and after collecting your data. California, United States: SAGE Publications, Inc.; 2013.

[21] Watkins MW. Exploratory factor analysis: a guide to best practice. J Black Psychol. 2018;44(3):219–46.

[22] Fabrigar LR, Wegener DT, Maccallum RC, Strahan EJ. Evaluating the use of Exploratory Factor Analysis in psychological research. Psychol Methods. 1999;4:272–99.

[23] Bandalos DL, Finney SJ. Factor Analysis 8. In: Factor Analysis The Reviewer’s Guide to Quantitative Methods in the Social Sciences. 2018. pp. 98–122.

[24] Izquierdo I, Olea J, Abad FJ. Exploratory factor analysis in validation studies: uses and recommendations. Psicothema. 2014;26(3):395–400. 25069561 10.7334/psicothema2013.349

[25] Hayes AF, Coutts JJ, But R. Use Omega rather than Cronbach’s alpha for estimating reliability. But… Commun Methods Meas. 2020;14(1):1–24.

[26] Brown TA. Confirmatory Factor Analysis for Applied Research. 2014.

[27] StataCorp. Stata Statistical Software: release 16. College Station. TX: StataCorp LLC; 2019.

[28] R Core Team. R: A language and environment for statistical computing. [Internet]. R Foundation for Statistical Computing, Vienna, Austria. 2021. Available from: https://www.r-project.org/

[29] Rosseel Y. Lavaan: an R Package for Structural equation. J Stat Softw. 2012;48(2).

[30] Revelle W. psych: Procedures for Personality and Psychological Research [Internet]. Northwestern University, Evanston, Illinois, USA; 2022. Available from: https://cran.r-project.org/package=psych

[31] Steiner MD, Grieder S. EFAtools: an R package with fast and flexible implementations of exploratory factor analysis tools. J Open Source Softw. 2020;5(2019):1–4.36756303

[32] Widaman KF, Revelle W. Thinking thrice about sum scores, and then some more about measurement and analysis. Behav Res Methods. 2023;55(2):788–806. 35469086 10.3758/s13428-022-01849-w PMC10027776

[33] Distefano C, Zhu M, Mîndrilã D. Understanding and Using Factor Scores: Considerations for the Applied Researcher. Practical Assessment, Research, and Evaluation. 2009;14:20.

[34] Downe S, Finlayson K, Oladapo O, Bonet M, Gülmezoglu AM. What matters to women during childbirth: a systematic qualitative review. Volume 13. PLoS ONE. Public Library of Science;; 2018. 10.1371/journal.pone.0194906 PMC5903648 29664907

[35] World Health Organization. WHO recommendations: Intrapartum care for a positive childbirth experience. Geneva; 2018. 30070803

[36] Freedman LP, Ramsey K, Abuya T, Bellows B, Ndwiga C, Warren CE, et al. Defining disrespect and abuse of women in childbirth: a research, policy and rights agenda. Bulletin of the World Health Organization. Volume 92. World Health Organization; 2014. pp. 915–7. 10.2471/BLT.14.137869 PMC4264393 25552776

[37] Balde MD, Bangoura A, Diallo BA, Sall O, Balde H, Niakate AS, et al. A qualitative study of women’s and health providers’ attitudes and acceptability of mistreatment during childbirth in health facilities in Guinea. Reprod Health. 2017;14(1):1–13. 28086975 10.1186/s12978-016-0262-5 PMC5237275

[38] Bohren MA, Vogel JP, Tunçalp Ö, Fawole B, Titiloye MA, Olutayo AO, et al. By slapping their laps, the patient will know that you truly care for her: a qualitative study on social norms and acceptability of the mistreatment of women during childbirth in Abuja, Nigeria. SSM Popul Health. 2016;2:640–55. 28345016 10.1016/j.ssmph.2016.07.003 PMC5356417

[39] Dzomeku VM, Boamah Mensah AB, Nakua EK, Agbadi P, Lori JR, Donkor P. I wouldn’t have hit you, but you would have killed your baby: exploring midwives’ perspectives on disrespect and abusive care in Ghana. BMC Pregnancy Childbirth. 2020;20(1). 10.1186/s12884-019-2691-y PMC6945392 31906875

[40] Bohren MA, Vogel JP, Tunçalp Ö, Fawole B, Titiloye MA, Olutayo AO et al. Mistreatment of women during childbirth in Abuja, Nigeria: a qualitative study on perceptions and experiences of women and healthcare providers. Reprod Health. 2017;14(1). 10.1186/s12978-016-0265-2 PMC5240205 28095911

[41] Agyenim-Boateng A, Cameron H, Bemah Boamah Mensah A. Health professionals’ perception of disrespectful and abusive intrapartum care during facility-based childbirth in LMIC: a qualitative systematic review and thematic synthesis. Int J Afr Nurs Sci. 2021;15.

[42] Orpin J, Puthussery S, Davidson R, Burden B. Women’s experiences of disrespect and abuse in maternity care facilities in Benue State, Nigeria. BMC Pregnancy Childbirth. 2018;18(1). 10.1186/s12884-018-1847-5 PMC5992700 29879944

[43] Sripad P, Merritt MW, Kerrigan D, Abuya T, Ndwiga C, Warren CE. Determining a trusting environment for Maternity Care: a Framework based on perspectives of women, communities, Service Providers, and managers in Peri-urban Kenya. Front Glob Womens Health. 2022;3. 10.3389/fgwh.2022.818062 PMC9069110 35528311

[44] Tumlinson K, Britton LE, Williams CR, Wambua DM, Otieno Onyango D. Informal payments for family planning: prevalence and perspectives of women, providers, and health sector key informants in western Kenya. Sex Reprod Health Matters. 2021;29(1):1–17. 34590988 10.1080/26410397.2021.1970958 PMC8494287

[45] Nawab T, Erum U, Amir A, Khalique N, Ansari M, Chauhan A. Disrespect and abuse during facility-based childbirth and its sociodemographic determinants– A barrier to healthcare utilization in rural population. J Family Med Prim Care. 2019;8(1):239. 30911513 10.4103/jfmpc.jfmpc_247_18 PMC6396581

[46] Uluman M, Doğan CD. Comparison of factor score computation methods in factor analysis. Aust J Basic Appl Sci. 2016;10(18):143–51.

[47] Magunda A, Ononge S, Balaba D, Waiswa P, Okello D, Kaula H et al. Maternal and newborn healthcare utilization in Kampala urban slums: perspectives of women, their spouses, and healthcare providers. BMC Pregnancy Childbirth. 2023;23(1). 10.1186/s12884-023-05643-0 PMC10163708 37147565

[48] Hughes CS, Kamanga M, Jenny A, Zieman B, Warren C, Walker D et al. Perceptions and predictors of respectful maternity care in Malawi: a quantitative cross-sectional analysis. Midwifery. 2022;112. 10.1016/j.midw.2022.103403 35728299

