# Supplementary material for: Measuring responsiveness and respectful treatment in maternity care in sub-Saharan Africa: a questionnaire validation and development of a score
Source: BMC Pregnancy Childbirth. 2025 Mar 21;25:329. doi: 10.1186/s12884-025-07319-3 (PMC11927248; doi:10.1186/s12884-025-07319-3)
Supplement: Supplementary file 1 — Supplementary Material 1 [file 12884_2025_7319_MOESM1_ESM.docx]

## Supplementary Material 1

## Questions and responses from the responsiveness and respectful treatment questionnaire used in the ALERT study.

The response options shown here are before any data manipulation was performed.

| Question | Responses |
| --- | --- |
| Did the providers introduce themselves to you with their names when they first came to see you? | 0=No, never, 1=Yes, a few times, 2=Yes, most of the time, 3=Yes, all of the time |
| Did the providers call you by your name? | 0=No, never, 1=Yes, a few times, 2=Yes, most of the time, 3=Yes, all of the time |
| Did the providers at the hospital treat you with respect? | 0=No, never, 1=Yes, a few times, 2=Yes, most of the time, 3=Yes, all of the time |
| Did the providers at the hospital treat you in a friendly manner? | 0=No, never, 1=Yes, a few times, 2=Yes, most of the time, 3=Yes, all of the time |
| How would you describe the waiting time before you were admitted to the labour ward? | 0=Very short, 1=Just a little long, 2=Somewhat long, 3=Very long |
| Were you slapped or pinched by a provider? | 0=No, 1=Yes, 2=Don't want to say, 3=Don't know |
| Were you held down to the bed forcefully by a provider? | 0=No, 1=Yes, 2=Don't want to say, 3=Don't know |
| Did you have forceful downwards pressure placed on your abdomen before the baby came out? | 0=No, 1=Yes, 2=Don't want to say, 3=Don't know |
| Were you shouted or screamed at by a provider or other member of staff? | 0=No, 1=Yes, 2=Don't want to say, 3=Don't know |
| Were you mocked at by a provider or other member of staff? | 0=No, 1=Yes, 2=Don't want to say, 3=Don't know |
| Did a provider make any negative comments e.g., about age/marital status/ethnicity/religion/HIV status? | 0=No, 1=Yes, 2=Don't want to say, 3=Don't know |
| Were you shouted at or told off because you did not bring items with you? | 0=No, 1=Yes, 2=Don't want to say, 3=Don't know |
| My private or personal information was shared without my consent | 0=No, never, 1=Yes, a few times, 2=Yes, most of the time, 3=Yes, all of the time |
| My physical privacy was violated e.g., being uncovered or having people in the delivery room without my consent | 0=No, never, 1=Yes, a few times, 2=Yes, most of the time, 3=Yes, all of the time |
| Did you feel the providers explained to you what had been done to you? | 0=No, never, 1=Yes, a few times, 2=Yes, most of the time, 3=Yes, all of the time |
| Did the provider ask you for permission before carrying out a vaginal examination? | 0=No, never, 1=Yes, a few times, 2=Yes, most of the time, 3=Yes, all of the time, 4=No vaginal examination was done |
| During labour and childbirth, did you feel like you were able to be in the position of your choice? | 0=No, never, 1=Yes, a few times, 2=Yes, most of the time, 3=Yes, all of the time |
| Did the provider speak to you in a language you could understand? | 0=No, never, 1=Yes, a few times, 2=Yes, most of the time, 3=Yes, all of the time |
| Did the provider explain to you why they were carrying out examinations or procedures? | 0=No, never, 1=Yes, a few times, 2=Yes, most of the time, 3=Yes, all of the time |
| Did the provider explain to you why they were giving you any medicine? | 0=No, never, 1=Yes, a few times, 2=Yes, most of the time, 3=Yes, all of the time, 4=No medication was prescribed/given |
| Did you feel you could ask the provider at the hospital any questions you had? | 0=No, never, 1=Yes, a few times, 2=Yes, most of the time, 3=Yes, all of the time |
| Did the provider at the hospital talk to you about how you were feeling? | 0=No, never, 1=Yes, a few times, 2=Yes, most of the time, 3=Yes, all of the time |
| Did the provider at the hospital address your anxieties and fears? | 0=No, never, 1=Yes, a few times, 2=Yes, most of the time, 3=Yes, all of the time, 4=I did not have any anxieties or fears |
| Were you encouraged to walk around during labour? | 0=No, never, 1=Yes, a few times, 2=Yes, most of the time, 3=Yes, all of the time, 4=Not relevant |
| Were you encouraged to eat and drink during labour? | 0=No, never, 1=Yes, a few times, 2=Yes, most of the time, 3=Yes, all of the time, 4=I didn't want to eat or drink |
| When you needed help, did you feel the providers at the hospital paid attention? | 0=No, never, 1=Yes, a few times, 2=Yes, most of the time, 3=Yes, all of the time, 4=I didn't need help |
| Did you feel providers helped you with your pain? Which statement describes this? | 0=I experienced pain, but I was not distressed and did not need any treatment, 1=I experienced pain that was distressing to me, and I received treatment that helped me to cope, 2=I experienced pain that was distressing to me, but did not receive any treatment, 3=I did not experience any pain |
| Were you allowed to have someone you wanted (family/friend) to stay with you during labour and contractions (1st stage of labour, the time before pushing)? | 0=No, never, 1=Yes, a few times, 2=Yes, most of the time, 3=Yes, all of the time, 4=I did not want someone to stay with me |
| Were you allowed to have someone you wanted (family/friend) to stay with you during birth (2nd stage of labour, pushing)? | 0=No, never, 1=Yes, a few times, 2=Yes, most of the time, 3=Yes, all of the time, 4=I did not want someone to stay with me |
| Did you feel the providers at the hospital took the best care of you that they could? | 0=No, never, 1=Yes, a few times, 2=Yes, most of the time, 3=Yes, all of the time |
| Did any of the providers or other staff suggest or ask you (or your family) for a bribe, informal payment or gift? | 0=No, 1=Yes, 2=Don't want to say, 3=Don't know |
| Would you say the hospital was clean? | 0=Very dirty, 1=Dirty, 2=Clean, 3=Very clean |
| Was there clean water in the hospital, e.g., for taking a shower? | 0=No, never, 1=Yes, a few times, 2=Yes, most of the time, 3=Yes, all of the time |
| Were you able to access clean drinking water whenever you needed it? | 0=No, never, 1=Yes, a few times, 2=Yes, most of the time, 3=Yes, all of the time |
| In general, did you feel safe in the hospital? | 0=No, never, 1=Yes, a few times, 2=Yes, most of the time, 3=Yes, all of the time |
| Would you recommend a family member to give birth in the same hospital? | 1=Very strongly recommend, 2=Strongly recommend, 3=Recommend, 4=Undecided, 5=Do not recommend, 6=Strongly do not recommend, 7=Very strongly do not recommend |
