# Supplementary material for: Measuring responsiveness and respectful treatment in maternity care in sub-Saharan Africa: a questionnaire validation and development of a score
Source: BMC Pregnancy Childbirth. 2025 Mar 21;25:329. doi: 10.1186/s12884-025-07319-3 (PMC11927248; doi:10.1186/s12884-025-07319-3)
Supplement: Supplementary file 2 — Supplementary Material 2 [file 12884_2025_7319_MOESM2_ESM.docx]

## Supplementary Material 2

## Data modification decisions.

Modifications were made to the data as responses needed to be able to be placed on a continuum of good/desirable responses to bad/undesirable responses to be able to perform the psychometric analyses. Not applicable (N/A) categories such as “Don’t want to say” or “Don’t know” cannot be placed on such a scale. Decisions on what to do with questions which have such categories were made in a research group meeting and are detailed below, along with the original responses from the questionnaire.

| Question | Original responses | Decision |
| --- | --- | --- |
| Were you slapped or pinched by a provider? | 0=No, 1=Yes, 2=Don't want to say, 3=Don't know | Categories 2 & 3 imputed onto median of categories 0 & 1 (non-N/A responses) |
| Were you held down to the bed forcefully by a provider? | 0=No, 1=Yes, 2=Don't want to say, 3=Don't know | Categories 2 & 3 imputed onto median of categories 0 & 1 (non-N/A responses) |
| Did you have forceful downwards pressure placed on your abdomen before the baby came out? | 0=No, 1=Yes, 2=Don't want to say, 3=Don't know | Categories 2 & 3 imputed onto median of categories 0 & 1 (non-N/A responses) |
| Were you shouted or screamed at by a provider or other member of staff? | 0=No, 1=Yes, 2=Don't want to say, 3=Don't know | Categories 2 & 3 imputed onto median of categories 0 & 1 (non-N/A responses) |
| Were you mocked at by a provider or other member of staff? | 0=No, 1=Yes, 2=Don't want to say, 3=Don't know | Categories 2 & 3 imputed onto median of categories 0 & 1 (non-N/A responses) |
| Did a provider make any negative comments e.g., about age/marital status/ethnicity/religion/HIV status? | 0=No, 1=Yes, 2=Don't want to say, 3=Don't know | Categories 2 & 3 imputed onto median of categories 0 & 1 (non-N/A responses) |
| Were you shouted at or told off because you did not bring items with you? | 0=No, 1=Yes, 2=Don't want to say, 3=Don't know | Categories 2 & 3 imputed onto median of categories 0 & 1 (non-N/A responses) |
| Did the provider ask you for permission before carrying out a vaginal examination? | 0=No, never, 1=Yes, a few times, 2=Yes, most of the time, 3=Yes, all of the time, 4=No vaginal examination was done | Category 4 imputed onto median of categories 0-3 (non-N/A responses) |
| Did the provider explain to you why they were giving you any medicine? | 0=No, never, 1=Yes, a few times, 2=Yes, most of the time, 3=Yes, all of the time, 4=No medication was prescribed/given | Category 4 imputed onto median of categories 0-3 (non-N/A responses) |
| Did the provider at the hospital address your anxieties and fears? | 0=No, never, 1=Yes, a few times, 2=Yes, most of the time, 3=Yes, all of the time, 4=I did not have any anxieties or fears | Though category 4 is an N/A response we kept it as the highest as it was decided that having no anxieties & fears was the most desirable situation |
| Were you encouraged to walk around during labour? | 0=No, never, 1=Yes, a few times, 2=Yes, most of the time, 3=Yes, all of the time, 4=Not relevant | Category 4 imputed onto median of categories 0-3 (non-N/A responses) |
| Were you encouraged to eat and drink during labour? | 0=No, never, 1=Yes, a few times, 2=Yes, most of the time, 3=Yes, all of the time, 4=I didn't want to eat or drink | Category 4 imputed onto median of categories 0-3 (non-N/A responses) |
| When you needed help, did you feel the providers at the hospital paid attention? | 0=No, never, 1=Yes, a few times, 2=Yes, most of the time, 3=Yes, all of the time, 4=I didn't need help | Combined categories 4 & 3 as decided it was best practice if they didn’t need help |
| Did you feel providers helped you with your pain? Which statement describes this? | 0=I experienced pain, but I was not distressed and did not need any treatment, 1=I experienced pain that was distressing to me, and I received treatment that helped me to cope, 2=I experienced pain that was distressing to me, but did not receive any treatment, 3=I did not experience any pain | Combined categories 0 & 3 as these both mean that no pain relief was required, and rearranged so that this was the middle response: 0=I experienced pain that was distressing to me, but did not receive any treatment, 1=I did not need any treatment for pain, 2=I experienced pain that was distressing to me, and I received treatment that helped me to cope |
| Were you allowed to have someone you wanted (family/friend) to stay with you during labour and contractions (1st stage of labour, the time before pushing)? | 0=No, never, 1=Yes, a few times, 2=Yes, most of the time, 3=Yes, all of the time, 4=I did not want someone to stay with me | Category 4 imputed onto median of categories 0-3 (non-N/A responses) |
| Were you allowed to have someone you wanted (family/friend) to stay with you during birth (2nd stage of labour, pushing)? | 0=No, never, 1=Yes, a few times, 2=Yes, most of the time, 3=Yes, all of the time, 4=I did not want someone to stay with me | Category 4 imputed onto median of categories 0-3 (non-N/A responses) |
| Did any of the providers or other staff suggest or ask you (or your family) for a bribe, informal payment or gift? | 0=No, 1=Yes, 2=Don't want to say, 3=Don't know | Categories 2 & 3 imputed onto median of categories 0 & 1 (non-N/A responses) |
