# Supplementary material for: Measuring responsiveness and respectful treatment in maternity care in sub-Saharan Africa: a questionnaire validation and development of a score
Source: BMC Pregnancy Childbirth. 2025 Mar 21;25:329. doi: 10.1186/s12884-025-07319-3 (PMC11927248; doi:10.1186/s12884-025-07319-3)
Supplement: Supplementary file 3 — Supplementary Material 3 [file 12884_2025_7319_MOESM3_ESM.docx]

## Supplementary Material 3

## Dates of data collection rounds.

| Country | Round 1 - baseline | Round 2 | Round 3 |
| --- | --- | --- | --- |
| Benin | 5^th^ December 2021 – 24^th^ January 2022 | 17^th^ June 2022 – 21^st^ July 2022 | 13^th^ December 2022 – 31st January 2023 |
| Malawi | 3^rd^ December 2021 – 22^nd^ December 2021 | 13^th^ August 2022 – 9^th^ September 2022 | 13^th^ February 2023 – 5^th^ March 2023 |
| Tanzania | 9^th^ December 2021 – 22^nd^ December 2021 | 18^th^ July 2022 – 6^th^ August 2022 | 8^th^ March 2023 – 31^st^ March 2023 |
| Uganda | 28^th^ January 2022 –16^th^ April 2022 | 8^th^ June 2022 – 11^th^ August 2022 | 17^th^ December 2022 – 7^th^ April 2023 |
